# ZnO-Impregnated Polyacrylonitrile Nanofiber Filters against Various Phases of Air Pollutants

**DOI:** 10.3390/nano11092313

**Published:** 2021-09-06

**Authors:** Hanaa Aamer, Sang-Bum Kim, Jong-Min Oh, Hyeokjin Park, Young-Min Jo

**Affiliations:** 1 Department of Environmental Science and Engineering, Kyung Hee University, Yongin 17104, Korea; aamerhanaa@gmail.com (H.A.); jmoh@khu.ac.kr (J.-M.O.); 2 Green Process and Material R&D Group, Korea Institute of Industrial Technology, Cheonan 31056, Korea; sbkim@kitech.re.kr

**Keywords:** nanofiber, composite, zinc oxide, particle dispersion, filtration, photocatalytic activity, antibacterial activity

## Abstract

The incorporation of metal oxide nanoparticles (NPs) in fiber filters is an effective approach to enhance the specific surface area and surface roughness of the fiber, hence improving their efficiency for fine dust capture and other gas treatment or biological applications. Nevertheless, uneven distribution of NPs limits their practical applications. In this study, a commercial silane coupling agent (3-methacryloxypropyltrimethoxysilane) was used to improve the dispersion of zinc oxide (ZnO) NPs in thin polyacrylonitrile fibers. Scanning electron microscopy (SEM) revealed that the fibers incorporating the silane-modified NPs exhibited better distribution of NPs than those prepared with pristine ZnO NPs. The silane modification enhanced the specific surface area, surface roughness, and fiber porosity. In particular, the nanofiber filter incorporating 12 wt% ZnO NPs modified with 0.5 g silane per g of ZnO NPs maintained a filtration efficiency of 99.76% with a low pressure drop of 44 Pa, excellent antibacterial activity, and could decompose organic methylene blue dye with an efficiency of 85.11% under visible light.

## 1. Introduction

Particulate matter (PM) pollution is a major risk that threatens human health in many regions of the world (WHO 2021). Atmospheric PM can be a carrier of bio-aerosols such as viruses, bacteria, and fungi [1,2], along with attached volatile organic compounds (VOCs). In particular, PM_1.0_ (i.e., particulate matter smaller than 1 micrometer) has gained much interest due to its ability to penetrate deep into the human lungs and interact with lung or bronchial cells. Inhalation of PM can cause heart disease, lung cancer, chronic and acute respiratory disease such as asthma, and infections due to carried viruses and bacteria [3]. Recent studies have reported that the main pathway of COVID-19 infection is the inhalation of airborne respiratory droplets and aerosols [4,5]. Therefore, protection against fine particulate matter and its associated pollutants has become the focus of recent research [6,7].

Electrospun nanofiber filters have been widely used in air cleaning applications as they can be woven into a systematic structure having a large specific surface area and high porosity that enable the effective capture of PM with low flow resistance [6,8,9]. Electrospun nanofibers have been fabricated from various polymers, including polyacrylonitrile (PAN), nylon, and polyvinylidene fluoride [10,11]. Among them, PAN is the most commonly used polymer due to its high dipole moment and excellent mechanical stability [12,13]. To meet the requirements for high-performance PM filtration, electrospun nanofibers can be modified by several structural and chemical methods [14], with one of the most effective being the combination of two or more materials with complementary functions [11]. For instance, a core–shell structured nanofiber filter composed of triphenyl phosphate core and nylon-6 shell has been shown to exhibit an excellent PM_2.5_ filtration efficiency and flame resistance property [15]. In addition, nanofibers incorporating metal oxide additives have recently gained increasing research interest [14]. Metal oxide additives can endow the nanofiber with various beneficial properties, including an increased specific surface area and, hence, an increased probability of PM impacting the fiber. Additionally, metal oxides create a rough fiber surface that can prevent particles from disentangling, thus resulting in enhanced filtration [6]. Furthermore, the metal oxides can impart additional functions such as photocatalytic activity [16] and antibacterial activity [17] to the fibers. For instance, the incorporation of TiO_2_ nanoparticles into polysulfone (PSU) formed nanoprotrusions on the fiber surface with micro- and nanoscale roughness that improved the filtration performance of the composite PSU/TiO_2_ fibers [18].

Zinc oxide (ZnO) is a multifunctional semiconductor oxide with excellent properties such as wide band gap (3.37 eV), high UV absorbance, non-toxicity, and excellent chemical stability [19]. In addition, as the safety of ZnO is acknowledged by the U.S. Food and Drug Administration (21CFR182.8991) [20], it is commonly used as an antibacterial agent for increasing the shelf-life of various products, and in textiles that come into direct contact with the human body [21]. Furthermore, ZnO has been reported to have excellent photocatalytic properties and antibacterial activity. For instance, Ghanbari et al. investigated the photocatalytic activity of ZnO nanorods, grown on the surface of alumina microfiber for liquid-phase and gas-phase decompositions of methylene blue and toluene, respectively. The results showed that ZnO nanorods grown on alumina could decompose the organics completely under UV irradiation within 10–20 min of light exposure [22]. The antibacterial activity of PAN nanofiber impregnated with ZnO nanoparticles has been investigated, and the PAN/ZnO composite was shown to have superior antibacterial activity against both *E. coli* and *S. aureus* [17].

A serious issue that is usually encountered during the incorporation of NPs into nanofibers is the ready agglomeration of the NPs, which leads to their inhomogeneous dispersion in organic solvents and, hence, poor distribution in the nanofiber structure [23]. In this case, the effect of the NPs cannot be fully released due to the shielding effect of the outer particles upon the inner particles of the agglomerate [24], leading to a negative effect on the pressure drop due to the significant impacts on fiber porosity and interconnectivity. Therefore, it is critical to prevent agglomeration in order to make the best use of the NPs [25]. In this respect, an effective approach is the surface modification of NPs by reaction with silane coupling agents [23]. The modification aims to enhance the interfacial interaction between polymers and inorganic NPs. Silanes are silicon-based materials that contain both inorganic and organic functional groups in the same molecule, therefore they can function as a connection bridge between organic and inorganic materials [26]. For example, the modification of ZnO NPs has been performed using 3-methacryloxypropyltrimethoxysilane (MPTMS) to improve their dispersion in polystyrene. The results indicated a homogeneous, monodisperse distribution of nanosized ZnO NPs within the composite, which resulted in enhanced thermal stability and UV absorption capacity compared to those of the pure polymer [27].

Following on from these past studies, the present work is aimed at achieving a good distribution with minimum agglomeration of the ZnO NPs in the nanofiber via the pre-modification with MPTMS as the coupling agent. The effects of this modification upon the fiber morphology and upon the PM filtration performance, photocatalytic activity, and antibacterial activity are investigated.

## 2. Materials and Methods

### 2.1. Materials

Polyacrylonitrile (PAN, Mw = 150,000) was purchased from Sigma-Aldrich, Co. (St. Louis, MO, USA). Zinc oxide nanoparticles (ZnO NPs, 99% purity) were supplied by Nanjing XFNANO Materials Tech Co. (Beijing, China). The commercial silane coupling agent, 3-methacryloxypropyltrimethoxysilane (MPTMS, 97% purity), and Methylene Blue (MB, 1% wt/vol aqueous solution) were procured from Alfa Aesar Chemical Co. (Seoul, Korea). Dimethylformamide (DMF, 99.8% purity) and potassium chloride (KCl, >99% purity) were obtained from Daejung Chemicals & Metals Co. (Seoul, Korea). Ethyl alcohol (99.9%, anhydrous) was supplied by Samchun Pure Chemical Co., LTD (Seoul, Korea). All reagents were used as received without further purification.

### 2.2. Modification of ZnO NPs with MPTMS

**Figure 1:**
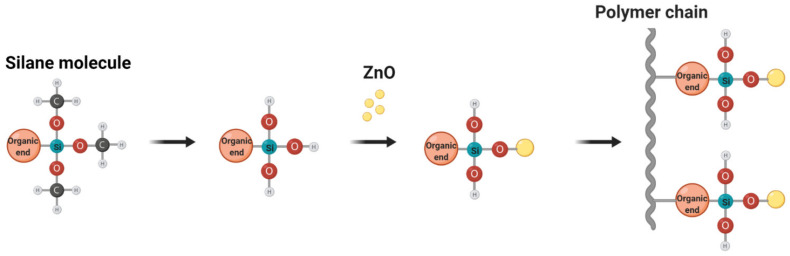
Schematic diagram of ZnO modification process with MPTMS.

The modification of ZnO NPs was performed via reaction with the silane coupling agent MPTMS, as shown schematically in Figure 1. First, 0.5, 1, and 2 g of silane were each added dropwise to 100 mL of ethanol, and the mixtures were stirred for 1 h to ensure full hydrolysis of the silane molecules. The amount of silane coupling agents used to modify ZnO NPs was selected based on the amounts reported in previous studies [23,25]. Subsequently, 1 g of pristine ZnO NPs (denoted as Z) was added to each of the three solutions, and stirring was continued for an additional 5 h. After that, the suspensions were subjected to sonication for 90 min to improve ZnO NP dispersion. The solution was then centrifuged, and the particles were separated from the liquid by decantation. The modified particles were then washed three times with ethanol to remove any excess silane, and oven dried at 90 °C for 8 h. All modification steps were performed in closed vials or glass beakers covered with aluminum foil and sealed tightly with plastic wraps to prevent the contamination of the surrounding atmosphere with chemicals. Thus, three types of ZnO NP were produced and are designated hereafter as Z(0.5S), Z(1S), and Z(2S), respectively. These were subsequently used as additives to PAN in the nanofiber preparation process.

### 2.3. Preparation of Electrospinning Solutions

Based on the results of our previous work [12], two ZnO concentrations in the nanofibers were selected for the present study, namely: 9 wt% and 12 wt%. As detailed in Table 1, a total of eight 10 mL test solutions were prepared, in which the solid content (including PAN and ZnO NPs) was held constant at 10 wt%. In each case, the selected amount of PAN was added to 9.54 mL of DMF, and the mixtures were stirred for 4 h. After complete dissolution of the PAN, the ZnO NPs were added and stirring was continued for an additional 20 h. Subsequently, the solutions were well dispersed in a sonic bath for 4 h to ensure homogeneity.

### 2.4. Nanofiber Fabrication by Electrospinning

An electrospinning technique was used to fabricate the nanofiber filters. The prepared electrospinning solutions were loaded into 10 mL syringes with a needle with an inner diameter of 0.337 mm. The syringe was driven by a syringe pump with a flow rate of 1 mL/h. A high voltage, 20 kV, was applied between the needle and the collector. The collector was a flat aluminum sheet covered with polypropylene substrate with dimensions 10 cm × 10 cm, located 15 cm away from the needle tip. The experimental set-up was enclosed in an isolated chamber measuring 60 cm × 50 cm × 50 cm. The temperature and humidity inside the chamber were controlled at 20–25 °C and 40–50%, respectively. After electrospinning, the nanofiber sheets were left to dry at an ambient condition for 2 days to ensure sufficient evaporation of the solvent.

### 2.5. Characterization of Nanofiber Filters

The fiber morphology was examined via field emission scanning electron microscopy (FE-SEM, LEOSUPRA 55, Carl Zeis Co., Krefeld, Germany). The SEM images were then analyzed using ImageJ software (ImageJ 1.x, LOCI, University of Wisconsin, Madison, WS, USA). The chemical characterization of the nanofiber filters was performed via energy-dispersive X-ray (EDX) spectroscopy to evaluate the presence and distribution of NPs within the fibers. The surface area and porosity of the fibers were analyzed by the nitrogen adsorption–desorption isotherms obtained at 77 K (BELSORP-mini II, MicrotracBEL, Osaka, Japan).

### 2.6. Filtration Test

**Figure 2:**
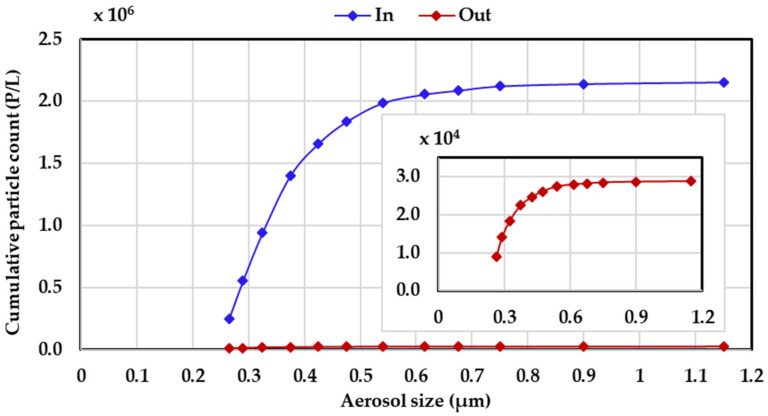
Particle size distribution of test particles before and after passage through the filter.

The filtration performance of the nanofiber filters was evaluated using a lab-built set-up. The test aerosol was generated by atomizing a 0.5 mol/L aqueous solution of potassium chloride (KCl), and dried by passing through a silica gel bed before being directed to the filter. A circular filter with a diameter of 5 cm was fixed inside the filter holder. The pressure drop across the filter was measured using a differential pressure manometer (Ulfa Technology Co., Ltd., Seoul, Korea). The number concentration according to particle size was measured before and after passage through the filter using two particle spectrometers (Grimm 11-A, Grimm Co., Ainring, Germany). The face velocity for all filtration tests was 5.3 cm/s. The test particle size of the inlet KCl aerosol was distributed between 0.265 to 1 µm, as shown in Figure 2. The filtration efficiency (η) was calculated based Equation (1).

(1) $$\eta =1-\frac{C_{\mathrm{down}}}{C_{\mathrm{up}}}$$

where C_up_ is the particle number concentration in the upstream position and C_down_ is the number concentration in the downstream. The pressure drop (ΔP) across the filter was measured using an electric differential manometer. The overall performance and quality factor (Q_f_) (Equation (2)) of the filter was then determined from Equation (2):

(2) $$Q_{f}=\frac{-\ln\left(1-\eta \right)}{P}$$

### 2.7. Photocatalytic Activity Test

**Figure 3:**
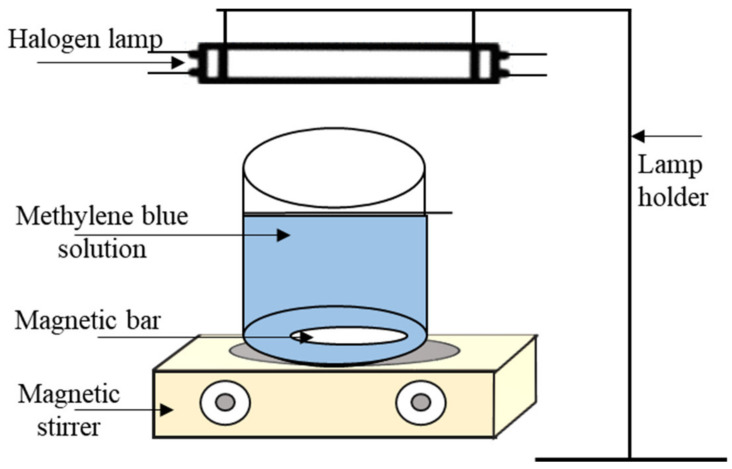
A schematic diagram of the photocatalytic activity test set-up.

The photocatalytic activity of the composite nanofiber filters was investigated by the degradation of methylene blue (MB) which is a highly colored, non-biodegradable, and toxic organic dye. All tests were performed under visible light irradiation (Haloline Eco 64,702,220 V 400 W R7s Osram, Seoul, Korea) using the experimental set-up shown in Figure 3. First, the fiber photocatalyst (25 mg) was added to MB solution (50 mL, 10 ppm) in a 100 mL beaker. The beaker was then completely covered with aluminum foil and the mixture was stirred in the dark for 30 min to achieve adsorption–desorption equilibrium [28] between the fiber and MB solution. After that, stirring was continued while the cover was removed and the light source turned on. Samples were then taken every 10 min for a period of 1 h. The samples were analyzed using a UV-Vis spectrophotometer (CARY 300 Bio, Agilent Tech., Santa Clara, CA, USA) at a wavelength of 664 nm.

### 2.8. Antibacterial Activity Test

The antibacterial activity was evaluated via a disk diffusion method. An agar solution was poured into a sterile culture dish and allowed to solidify. Afterwards, 100 µL of Gram-negative *E. coli* bacterial suspension was distributed uniformly in the dish. Circular nanofiber films (8 mm in diameter) were cut and placed on the surface of the bacterial suspension using sterile forceps. After overnight incubation at 37 °C, the regions of inhibition were measured using a ruler.

## 3. Results and Discussion

Since sample preparation including ZnO NP impregnation with silane pretreatment is time-consuming work, five samples were repeatedly prepared for each condition, and the test results and characteristics were averaged.

### 3.1. Effect of Silane on ZnO Dispersion in Nanofibers

#### 3.1.1. Modified ZnO

**Figure 4:**
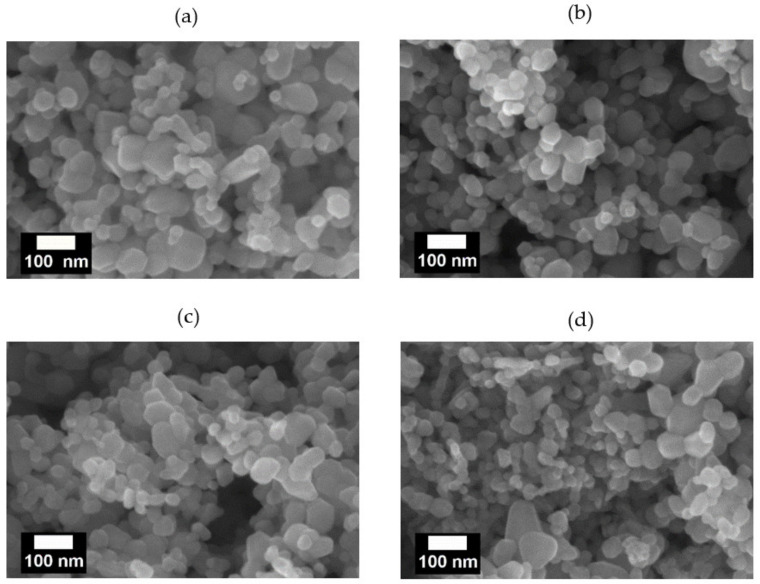
SEM images of (**a**) pristine ZnO NPs, (**b**) Z(0.5S), (**c**) Z(1S), and (**d**) Z(2S).

As detailed in the experimental section, the ZnO NPs were modified using a silane coupling agent to prevent their agglomeration, and hence improve their dispersion in the nanofibers. The SEM images of the pristine and modified ZnO NPs are presented in Figure 4, where the pristine ZnO NPs exhibit large aggregates with an average diameter of 54.65 nm and a wide size distribution (standard deviation = 28.29 nm) (Figure 4a, Table 2). By contrast, the average particle sizes of Z(0.5S), Z(1S), and Z(2S) samples are reduced to 40.82, 39.26, and 41.2 nm (Figure 4b–d), respectively, with Z(0.5S) exhibiting the most uniform distribution and Z(1S) presenting the broadest distribution. In addition, the silane-modified NPs exhibit more voids and spaces between the particles compared to the untreated ZnO NPs. These results demonstrate that modification with the silane coupling agent plays an effective role in preventing particle agglomeration.

#### 3.1.2. Morphological Characterization of PAN/ZnO (Silane) Nanofibers

**Figure 5:**
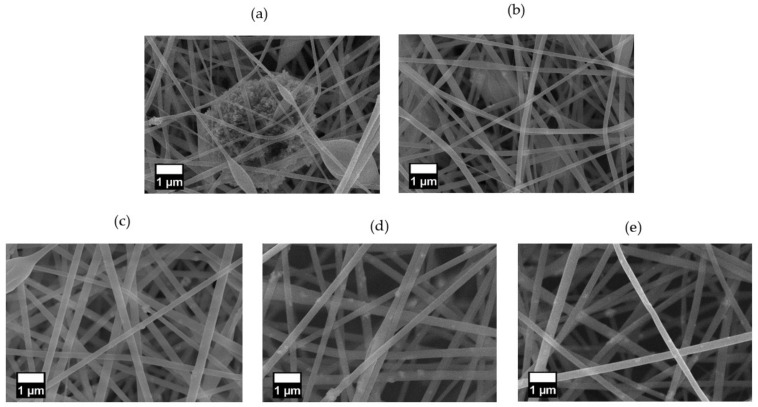
SEM images of (**a**,**b**) PZ9, (**c**) PZ9(0.5S), (**d**) PZ9(1S), and (**e**) PZ9(2S) nanofibers.

**Figure 6:**
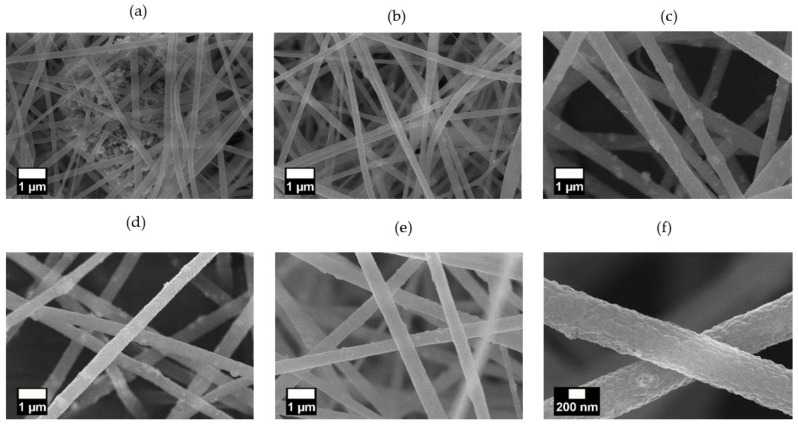
SEM images of (**a**,**b**) PZ12, (**c**) PZ12(0.5S), (**d**) PZ12(1S), and (**e**) PZ12(2S), and (**f**) highly magnified image of PZ12(0.5S) nanofibers.

The SEM images of the nanofibers prepared with the pristine ZnO NPs (PZ9 and PZ12) and silane-modified ZnO NPs (PZ(S)) are presented in Figure 5 and Figure 6, respectively. Here, both the PZ9 and PZ12 samples exhibit particle aggregates in and between nanofibers (Figure 5a and Figure 6a) including some regions of densely aggregated nanoparticles that form huge agglomerates, thus leading to inhomogeneous fiber characteristics. Several nanofiber morphologies, including thick and thin fibers, and irregular beads with various sizes, are observed around these aggregated NPs. It is anticipated that this morphological diversity within the same fiber might lower its mechanical strength, and greatly limit the utilization of the NPs. Elsewhere along the same nanofibers (Figure 5b and Figure 6b), only a small quantity of NPs is observed because agglomeration of the particles has occurred in other parts of the fiber. Hence, the morphology of this area is more uniform than that of the area containing the aggregated NPs. Nevertheless, the poor distribution of NPs within the more uniform area remains problematic because the ZnO NPs do not effectively perform their function.

By contrast, the level of particle agglomeration was seen to be gradually reduced with increasing silane content in the pre-modified ZnO NPs (Figure 5c–e), the distribution of the NPs in the nanofibers was greatly enhanced, and more uniform nanofibers with few or no beads were also produced. In addition, the distance between the nanofibers was seen to gradually increase as the amount of silane increases, thus potentially decreasing the pressure drop during fine dust filtration. Similar trends were observed for the PZ12 nanofibers with the pre-modified ZnO NPs in Figure 6c–e, with an obviously better distribution compared to the sample containing the unmodified ZnO NPs. In particular, the highly magnified image of the PZ12(0.5S) sample in Figure 6f reveals the rough surface of the fibers.

The porosity of the PZ12 nanofibers incorporating the modified ZnO NPs was also greatly enhanced compared to those containing the unmodified ZnO NPs under the same electrospinning conditions, with PZ12(0.5S) exhibiting the highest porosity. On the contrary, the opposite trend in porosity was observed for the PZ9 samples, with PZ9(2S) exhibiting the highest porosity. The results summarized in Table 3 indicate that the specific surface areas and pore volumes of the nanofiber samples were greatly enhanced by the silane modification. In particular, the specific surface area of the PZ9 sample was increased from 21 m^2^/g with the unmodified ZnO to 29 m^2^/g for PZ9(2S), accompanied by a pore volume increase from 0.058 cm^3^/g to 0.081 cm^3^/g. Meanwhile, the specific surface area of PZ12 was significantly increased from 24 m^2^/g with the unmodified ZnO to a maximum of 36 m^2^/g in PZ12(0.5S), while the total pore volume increased from 0.064 cm^3^/g to 0.099 cm^3^/g. In addition, mesopore volume preferable for photocatalytic activity was also large with 0.098 cm^3^/g in PZ12(0.5S). The presence of mesopores within the fibers is supposed to improve mass transport, and thereby to contribute to effective decomposition reactions inside, particularly under exciting light [29]. The pores of all samples were from 8.42 nm to 19.52 nm, and PZ12(0.5S) had the largest size with an average of 19.52 nm.

The different trend in specific surface area with the addition of silane for PZ12 compared to that of PZ9 could be attributed to the different ratios of ZnO NPs to silane. Thus, a suitable amount of silane is required for good dispersion of the ZnO NPs in the PAN solution and, hence, good interconnection between the ZnO NPs and the PAN in the composite structure. In the case of PZ12(2S), however, the presence of too much silane hindered the dispersion of the ZnO NPs in the polymer solution, as the silane groups tended to bind the ZnO NPs to each other rather than forming intermolecular links between the ZnO and PAN [24]. This analysis suggests that there is an optimum combination of ZnO and silane in the fiber polymer precursor composed of PAN.

#### 3.1.3. Chemical Characterization of PAN/ZnO(Silane) Nanofiber

The effects of silane modification upon the dispersion of ZnO NPs in the various nanofibers were further demonstrated by the weight percent of elemental Zn, as revealed by the EDX results in Table 4. Here, the zinc content of the PZ9 sample was seen to increase from only 4.81 wt% with the unmodified ZnO NPs to 6.02 wt% for PZ9(0.5S). Despite the having same amount of ZnO, the apparent concentration of Zn detected in the fibers could be different depending on the silane pretreatment, reaching 6.7 wt% for PZ9(2S). Similarly, the Zn concentration in the PZ12 nanofiber is increased significantly from 6.97 wt% with the unmodified ZnO NPs to 9.76 wt% for PZ12(0.5S). Further, although the Zn concentration subsequently decreased with an increasing amount of silane in the PZ12(1S) and PZ12(2S) samples, it remained higher than that of the unmodified PZ12. Thus, for a given amount of ZnO, the concentration of Zn found in the fibers depends on the silane pretreatment. This result further demonstrates that the silane coupling agent helps to disperse ZnO NPs in the polymer solution, and is consistent with the abovementioned specific surface area and porosity analyzed.

Although a larger amount of silane was expected to improve the distribution of NPs, as was observed for the PZ9 samples, the lower ratio of PAN to ZnO in the PZ12 nanofibers means that a larger number of NPs must be distributed in a smaller amount of polymer. This crowding of NPs into a smaller amount of fiber was further aggravated as the amount of silane was increased, so that the silane molecules tended to bind the ZnO NPs to each other rather than linking them to the polymer. It could be concluded that the modification with 0.5S was sufficient for good distribution of the ZnO NPs in the PZ12 fibers, whereas increasing the amount of silane promoted particle agglomeration. Therefore, excessive silane was not favorable for the even distribution of NPs.

### 3.2. Performance of PAN/ZnO (Silane) Fiber Filters for Air Pollutants

#### 3.2.1. Filtration Performance

To evaluate the effects of the silane-modified NPs on the fine dust capture performance, filters were prepared using each of the various nanofiber compositions. The electrospinning time was adjusted for each filter so that the test filters exhibited a consistent pressure drop of 20 Pa.

**Figure 7:**
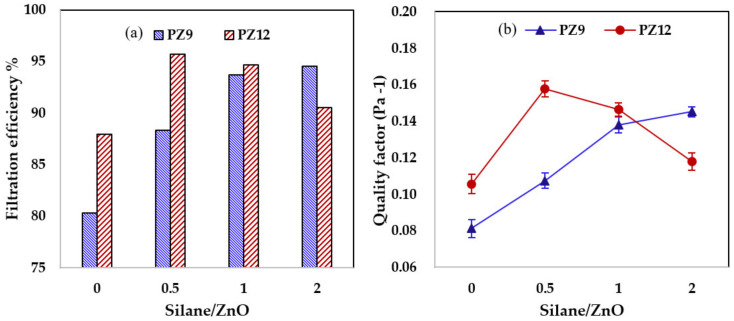
Filtration performance of various nanofiber filters for 300 nm KCl particles under a constant pressure drop of 20 Pa; (**a**) filtration efficiency and (**b**) quality factor.

The filtration performances of the various nanofiber filters for 300 nm KCl particles at a velocity of 5.3 cm/s were compared in Figure 7a. Here, the filtration efficiency of the PZ9 filter increased significantly from 80.33% with the unmodified ZnO NPs to 88.34% for PZ9(0.5S) and 94.53% for PZ9(2S). This increase in filtration efficiency with increased silane content was attributed to the concurrent increase in the specific surface area and porosity of the nanofibers. On the other hand, the filtration efficiency of the PZ12-based nanofiber filters initially increased with increasing silane content, up to a maximum of 95.73% for PZ12(0.5S), and decreased thereafter. This tendency is consistent with the chemical analysis by EDX and surface area. Moreover, similar trends are observed in the plots of quality factor against silane content in Figure 7b.

**Figure 8:**
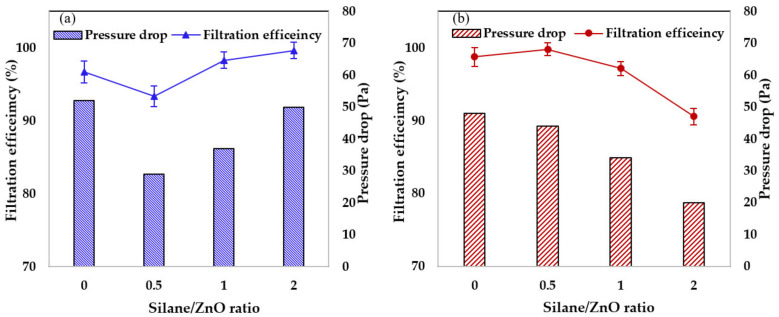
Filtration efficiencies and pressure drops of (**a**) PZ9-based filters and (**b**) PZ12-based filters.

Each filter was optimized to achieve the best combination of high filtration efficiency and low pressure drop. For each nanofiber type, thin filters were prepared by manipulating the electrospinning time, then the filtration efficiency and quality factor (Q_f_) were evaluated. For each type of filter, the pressure drop (bar chart, right-hand axis) and the corresponding filtration efficiency (graph, left-hand axis) are indicated in Figure 8. Here, the filtration efficiency of the PZ9 filter (Figure 8a) was seen to initially decrease from 96.64% with the unmodified ZnO NPs to 93.37% for PZ(0.5S), and then to increase with increasing silane content to 98.29% and 99.64% for PZ9(1S) and PZ9(2S), respectively. Similarly, the pressure drop initially decreased from 52 Pa for the unmodified PZ9 to 29 Pa for PZ(0.5S), and then increased to 37 Pa and 50 Pa for PZ9(1S) and PZ9(2S), respectively. By contrast, the filtration efficiency of the PZ12 filter initially increased from 98.8% with the unmodified ZnO NPs to 99.76% for PZ12(0.5S), and then decreased with the further addition of silane (Figure 8b). This trend was also reflected by the pressure drop.

Overall, the best filtration efficiency was observed for PZ9(2S) (99.64%) and PZ12(0.5S) (99.76%) with pressure drops of 50 and 44 Pa, respectively. The improvement of the filtration performance of the composite nanofiber filters with silane modification is further demonstrated by comparisons of the corresponding quality factor, which are listed in Table 5. The quality factor of PZ9 was seen to increase from 0.065 Pa^−1^ with the unmodified ZnO NPs to 0.112 Pa^−1^ for PZ9(2S), while that of PZ12 increased from 0.092 Pa^−1^ with unmodified ZnO NPs to 0.137 Pa^−1^ for PZ12(0.5S). This improvement in filtration performance could be attributed to the increased dust capture efficiency provided by the enhanced surface area and surface roughness, and the low pressure drop provided by the improved fiber porosity.

Table 5 also compares the filtration performance of these representative nanofiber filters with those of previously reported filters. Thus, a PAN/TiO_2_ bead/P25 nanofiber composite has been shown to provide a filtration efficiency of 96.75% and a pressure drop of 88 Pa [30] while the highest filtration efficiency of 99.99% was reported for a ZnO@PVA/KGM nanofiber filter with a pressure drop of 130 Pa [3]. With a similar test dust, the as-prepared PZ12(0.5S) exhibits a filtration efficiency of 99.76% with a low pressure drop of 44 Pa. This significant reduction in pressure drop while maintaining a high filtration efficiency explains why this filter exhibited the highest quality factor among the various reported PM filtration materials. Therefore, the PAN/ZnO composite nanofiber filter can be utilized in respiratory applications, providing excellent breathability, as well as in low-energy filtration applications.

**Figure 9:**
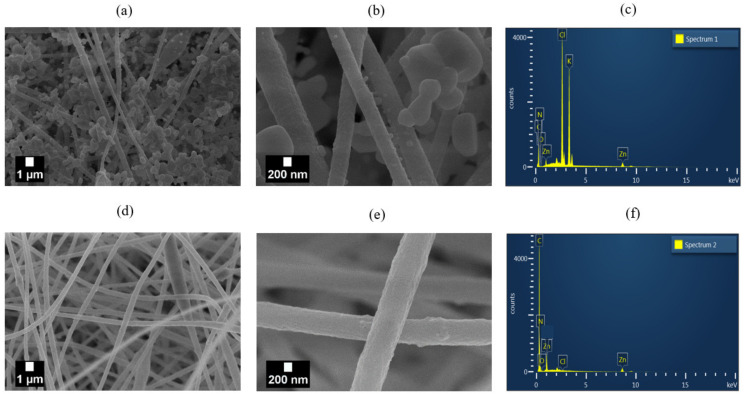
SEM images of PZ12(0.5S)-based filter: after filtration test (**a**,**b**), after washing with DW (**d**,**e**); EDX analyses are presented in (**c**,**f**), respectively.

The SEM images of the PZ12(0.5S) filter in Figure 9 were obtained after the filtration test (Figure 9a,b) and after cleaning with deionized water (Figure 9d,e). Thus, the filter is seen to have collected a large amount of test dust (Figure 9a), and an analysis of the high-magnification image in Figure 9b using ImageJ software indicates a wide range of captured particle sizes (from 20 nm to over 1 µm). The capture of nanosized particles thus demonstrates the potential ability of the filter to capture very small bio-aerosols such as bacteria and viruses. Furthermore, the EDX analysis of the filter with the captured particles (Figure 9c) clearly reveals the presence of high concentrations of K and Cl, while the complete removal of the filtered particles via the cleaning process is confirmed by the EDX analysis in Figure 9f.

#### 3.2.2. Photocatalytic Activity

**Figure 10:**
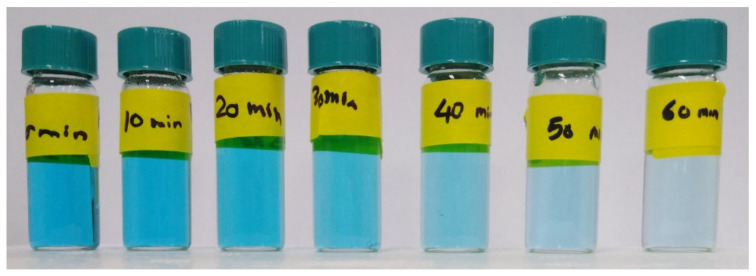
Change in MB solution color at 10 min intervals for 1 h due to photocatalytic decomposition under visible light irradiation.

**Figure 11:**
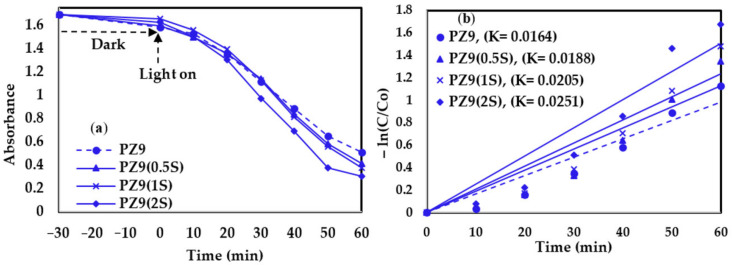
Photocatalytic degradation performance of various PZ9 fibers under visible light irradiation: (**a**) absorbance against time, and (**b**) ln(C/C_0_) against time (R^2^ of approximation: 0.89–0.92).

**Figure 12:**
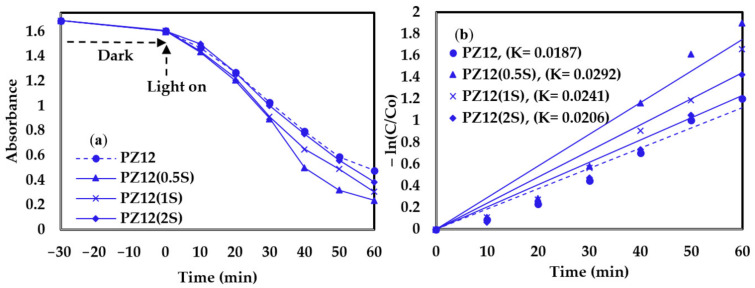
Photocatalytic degradation performance of various PZ12 fibers under visible light irradiation: (**a**) absorbance against time, and (**b**) ln(C/C_0_) against time (R^2^ of approximation: 0.93–0.96).

The photocatalytic activity of the various as-prepared nanofibers towards the degradation of MB dye is illustrated visually by the examples in Figure 10, and by the plots of absorbance against time and −ln(C/C_0_) against time for the PZ9- and PZ12-based nanofibers in Figure 11 and Figure 12, respectively. Here, all the filters exhibit very small reductions in the MB concentration under dark conditions, and significant reductions in the MB concentration upon illumination (Figure 11a and Figure 12a). Moreover, the results summarized in Table 6 indicate an increase in the decomposition efficiency from 67.65% and 70.12% for the unmodified PZ9 and PZ12, respectively, to maximum values of 81.30% for PZ9(2S) and 85.11% for PZ12(0.5S). These results highlight the importance of the good distribution of ZnO NPs provided by the silane modification for enhancing the photocatalytic effect of ZnO. Further, the relative concentration gradients in Figure 11b and Figure 12b indicate that the photocatalytic reaction follows the first-order rate law with rate constants (K) of 0.0164 min^−1^ and 0.0187 min^−1^ for the unmodified PZ9 and PZ12, respectively, and 0.0251 min^−1^ and 0.0292 min^−1^ for PZ9(2S) and PZ12(0.5S), respectively. These results demonstrate that the rate of the photocatalytic reaction was enhanced by the silane modification. This, in turn, can be attributed to the improvements in the surface area and pore volume of the nanofibers due to the good distribution of NPs. In brief, the modified nanofibers could achieve a high photocatalytic degradation efficiency of 85.11% under visible light irradiation, which is beneficial for the decomposition of organic pollutants under ambient conditions.

#### 3.2.3. Antibacterial Activity

**Figure 13:**
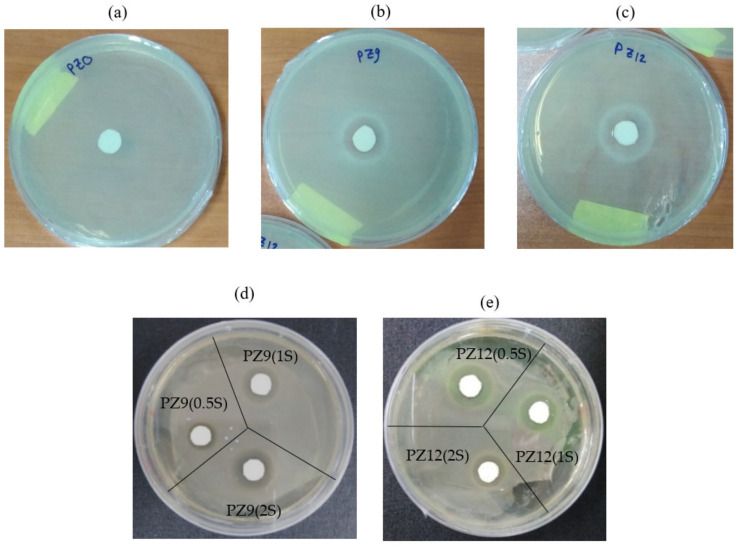
Zones of inhibition around (**a**) pure PAN nanofiber, (**b**) unmodified PZ9, (**c**) unmodified PZ12, (**d**) various PZ9(S) and (**e**) PZ12(S) nanofibers.

The antibacterial activities of pure PAN (designated as PZ0) and the various as-prepared composite nanofibers against Gram-negative *E. coli* bacteria are demonstrated in Figure 13. Here, the pure PAN nanofibers did not exhibit any antibacterial activity (Figure 13a), but 18 mm and 20 mm inhibition zones were observed around the PZ9 and PZ12 fibers, respectively (Figure 13b,c). The antibacterial activity of the unmodified ZnO-containing composite nanofibers might be attributed to the following mechanisms: (i) electrostatic interaction between the positively charged NPs and the negatively charged bacterial cells generates a high oxidative stress level that can destroy cell proteins, and/or (ii) reactive oxygen species (ROS) such as H_2_O_2_ or superoxide ions may be produced indirectly by the NPs, thus resulting in a severe oxidative stress and damage to the cell macromolecules, inhibition of enzymes, disruption of proteins, and damage to the RNA/DNA. Meanwhile, the effects of silane modification upon the antibacterial activities of the PZ9 and PZ12 fibers are demonstrated in Figure 13d,e, respectively. Here, the zone of inhibition around the PZ9-based fibers is seen to increase with increasing silane content, from 14 mm for PZ9(0.5S) to 20 mm for PZ9(2S) (Table 7). By contrast, PZ12(0.5S) shows the highest antibacterial activity among all the silane-modified PZ12 samples, with an inhibition zone of 23 mm. This may be attributed to an excessive amount of silane hindering the dispersion of the ZnO NPs throughout the fibers. Thus, despite an apparent antibacterial function, the optimum formulation of nanofiber filters should be carefully determined on the basis of various preliminary tests.

## 4. Conclusions

In this study, ZnO NPs were modified with a silane coupling agent to avoid their agglomeration in PAN nanofiber and, thus, ensure the homogenous distribution of NPs within the nanofiber. The nanofiber prepared with silane-modified ZnO NPs showed obvious improvement in specific surface area, surface roughness, and fiber porosity compared to the nanofiber prepared with pristine ZnO NPs. In addition, the nanofibers containing 12 wt% ZnO NPs modified with a silane to ZnO ratio of 0.5 achieved a fine particle filtration efficiency of 99.76% with a very low pressure drop of 44 Pa, and offered a high photocatalytic efficiency for decomposition of MB dye under visible light irradiation. Moreover, the PAN/ZnO nanofiber composite containing silane-modified ZnO NPs exhibited an excellent antibacterial activity against Gram-negative *E. coli* bacteria. The improvement in nanofiber surface characteristics, filtration performance, and photocatalytic activity with silane modification highlights the importance of the homogeneous distribution of the ZnO additive for optimizing the effects of the NPs.

## Figures and Tables

**Table 1:** Recipe of electrospinning solutions for PAN/ZnO silane composites.

Sample ID	Silane/ZnO Ratio in Mass	Relative Mass of ZnO to Total Solid Content (%)
PZ9	0	9
PZ9(0.5S)	0.5	9
PZ9(1S)	1	9
PZ9(2S)	2	9
PZ12	0	12
PZ12(0.5S)	0.5	12
PZ12(1S)	1	12
PZ12(2S)	2	12

Symbols of sample ID: P = PAN, Z = ZnO, S = silane.

**Table 2:** Particle sizes of pristine and modified ZnO NPs.

Sample	Particle Size (nm)	Standard Deviation (SD)
Z	54.65	28.29
Z(0.5S)	40.82	15.91
Z(1S)	39.26	23.75
Z(2S)	41.20	19.54

**Table 3:** Textural analysis of the test nanofibers.

Sample	A (m^2^/g)	V_p_ (Total) (cm^3^/g)	V_meso_ (cm^3^/g)	d_avg_ (nm)
PZ9	21	0.058	0.056	8.42
PZ9(0.5S)	22	0.061	0.060	8.55
PZ9(1S)	25	0.070	0.069	10.79
PZ9(2S)	29	0.081	0.079	12.32
PZ12	24	0.064	0.062	11.53
PZ12(0.5S)	36	0.099	0.098	19.52
PZ12(1S)	33	0.092	0.090	18.01
PZ12(2S)	27	0.075	0.072	17.18

A: specific surface area, V_p_ (total): total pore volume, V_meso_: mesopore volume, d_avg_: average pore size. All parameters were determined based on N_2_ adsorption-desorption isotherms.

**Table 4:** Elemental zinc (Zn) contents of the PZ9 and PZ12 samples according to EDX analyses.

Sample	Zn wt%
PZ9	4.81
PZ9(0.5S)	6.02
PZ9(1S)	6.41
PZ9(2S)	6.70
PZ12	6.97
PZ12(0.5S)	9.76
PZ12(1S)	8.62
PZ12(2S)	7.76

**Table 5:** Pressure drop, filtration efficiency, and quality factor values for the as-prepared and previously reported nanofiber filters.

Polymer or Polymer Composite	Test Aerosol (Size in nm)	Face Velocity (cm/s)	Filtration Efficiency (%)	Pressure Drop (Pa)	Quality Factor (Pa^−1^)	References
ZnO@PVA/KGM	NaCl (100)	5.3	99.99	130	0.0708	[3]
PAN/TiO_2_ beads/P25	NaCl (30–500)	NA	96.75	88	0.039	[30]
PEO	KCl (100)	5	94.1	90.42	0.031	[31]
Ag/PAN	NaCl (9–300)	5	98.65	68	0.05	[32]
PU/PET	NaCl (260)	5.3	76.63	17.3	0.084	[33]
PZ9	KCl (300)	5.3	96.64	52	0.065	This work
PZ9(2S)	KCl (300)	5.3	98.80	48	0.092	This work
PZ12	KCl (300)	5.3	99.64	50	0.112	This work
PZ(0.5S)	KCl (300)	5.3	99.76	44	0.137	This work

**Table 6:** Photocatalytic efficiency by various as-prepared filters towards degradation of MB under visible light irradiation.

Sample	Photocatalytic Degradation Efficiency (%)
PZ9	67.65
PZ9(0.5S)	73.98
PZ9(1S)	77.21
PZ9(2S)	81.30
PZ12	70.12
PZ12(0.5S)	85.11
PZ12(1S)	80.99
PZ12(2S)	75.99

**Table 7:** Zone of inhibition of nanofibers prepared with intrinsic and silane-modified ZnO NPs.

Sample	Zone of Inhibition (mm)
PZ0	0
PZ9	18
PZ9(0.5S)	14
PZ9(1S)	18
PZ9(2S)	20
PZ12	20
PZ12(0.5S)	23
PZ12(1S)	20
PZ12(2S)	17

## Data Availability

The data presented in this study are available on request from the corresponding author.
